# Poor Mobilization and Plerixafor Use in Matched Related Peripheral Hematopoietic Progenitor Cell Donors

**DOI:** 10.1002/jca.70074

**Published:** 2025-12-20

**Authors:** Elizabeth A. Godbey, Nicole L. Draper, Laura Connelly‐Smith, Caroline Alquist, Joseph Schwartz, Leonor Fernando, Andrew Jones, Amy E. Schmidt, Charles Harmon, Esther Lee, Yevgeniy Linnik, Laura Cooling

**Affiliations:** ^1^ Department of Laboratory Medicine and Pathology Mayo Clinic Jacksonville Florida USA; ^2^ Department of Pathology University of Colorado Anschutz Medical Campus Denver Colorado USA; ^3^ Fred Hutch Cancer Center Seattle Washington USA; ^4^ Hoxworth Blood Center, University of Cincinnati Cincinnati Ohio USA; ^5^ Foundation for the Accreditation of Cellular Therapy (FACT) Omaha Nebraska USA; ^6^ Department of Pathology and Laboratory Medicine University of California Davis Sacramento California USA; ^7^ Department of Laboratory Medicine UCSF Benioff Children's Hospital Oakland Oakland California USA; ^8^ HCA Denver Colorado USA; ^9^ Helena Laboratory Physicians/St. Peter's Health Helena Montana USA; ^10^ University of Michigan Medical School, University of Michigan Ann Arbor Michigan USA; ^11^ Department of Medical Affairs Labcorp Biopharma Indianapolis Indiana USA; ^12^ Department of Pathology University of Michigan Ann Arbor Michigan USA

**Keywords:** allogeneic hematopoietic donor, matched related donor, plerixafor

## Abstract

Plerixafor (PLX) is FDA approved for use in autologous peripheral blood stem cell donors but not in allogeneic donors. This study was completed by members of the ASFA HPC Donor Subcommittee to examine the incidence and characteristics of poor mobilizers (PM) among matched related donors (MRD), as well as factors associated with PLX use in MRD. Risks of poor mobilization in MRD were older age, especially donors older than 60 years, lower baseline platelet counts, and heavier recipients. PLX use in PM was low but safe, tripling the success rate for collection. This study adds evidence to the body of literature to support use of PLX in allogeneic donors who are PM.

## 1 Introduction

Plerixafor (PLX) is FDA approved for use in autologous peripheral blood stem cell donors but not in allogeneic donors. In autologous donors, PLX is commonly used for poorly mobilizing donors. This study was completed by members of the ASFA HPC Donor Subcommittee to examine the incidence and characteristics of poor mobilizers (PM) among matched related donors (MRD), as well as factors associated with PLX use in MRD.

## 2 Methods

A 4‐year (2014–2017) retrospective chart review was completed at 10 medical centers. Each center obtained IRB approval with waived informed consent for the study. Inclusion criteria included allogeneic MRD, documented day 1 CD34/kg yield and final CD34/kg yield. Collected data included CD34/kg yield, donor and recipient demographics (age, race, weight, BMI, obesity, and sex), peripheral blood counts (WBC, CD34, and platelet), mobilization agent (G‐CSF dose, PLX use, and peripheral blood counts), procedural details (machine type, number of procedures, blood volume processed, and anticoagulant), and CD34 yield. The MOBIL1 definitions for good mobilizers (GM; ≥ 2 × 10^6^ CD34/kg‐recipient in one procedure), PM (< 2 × 10^6^ CD34/kg) and successful mobilization (≥ 4.5 or ≥ 5 × 10^6^ CD34/kg) were utilized in analysis [1]. A successful mobilization of at least 5 × 10^6^ CD34/kg was the standard target yield for participating centers. A CD34 yield of at least 4.5 × 10^6^ CD34/kg was used in the MOBIL1 trial and by NMDP. Mean, standard deviation, *t* test, *χ* ^2^, odds ratios (OR), and 95% confidence intervals (CI) were performed using commercial software.

## 3 Results

Of the 432 MRD who met criteria for analysis, 39 were PM (9.0%) on day 1 collection (Table 1). For PM, day 1 mean CD34/μL was 28 (vs. 92, *p* < 0.0001) and day 1 CD34 yield was 1.3 × 10^6^/kg (vs. 8.8 × 10^6^/kg, *p* < 0.0001). There were no significant differences in G‐CSF dose, instrument, or blood volumes processed on day 1 (GM vs. PM). Overall, the PM group required more procedures (*p* < 0.0001) and total blood volumes processed (7.2 vs. 4.2 L, *p* < 0.0001) per mobilization, with a lower final CD34 yield (4.1 × 10^6^/kg) vs. GM (10.2 × 10^6^/kg, *p* < 0.0001).

**TABLE 1:** Comparison of matched related allogeneic HPC donors by CD34 yields on day 1 collection.

Variables	Mobilization response (Day 1 HPCC)		Good vs. Poor (P)	Mobilization by PLX usage		+ PLX vs. no PLX (P)
	Good (≥ 2 × 10^6^ CD34/kg)	Poor (< 2 × 10^6^ CD34/kg)		+ PLX	No PLX	
No. Donations	393 (91%)	39 (9%)	—	8	31	—
Donor characteristics						
Mean age, years	49.6 ± 13.1	55.9 ± 12.9	0.004	59.5 ± 7.5	55.1 ± 13.9	0.39
Age ≥ 60 years (%)	97 (25%)	19 (49%)	0.001 OR = 2.90 (CI: 0.17–0.67)	4 (50%)	15 (48%)	0.94
Sex						
Male	203 (52%)	15 (39%)	Reference	0	15 (48%)	Reference
Female	190 (48%)	24 (61%)	0.12	8 (100%)	16 (52%)	0.013
Weight, kg	87.6 ± 20.8	82.2 ± 18.5	0.09	74.9 ± 17.4	84.2 ± 18.6	0.21
BMI, kg/M^2^	30.1 ± 6.3	28.9 ± 6.5	0.25	28.1 ± 4.6	29.1 ± 6.9	0.71
Obese (BMI ≥ 30)	174 (44%)	11 (28%)	0.053	3 (37%)	8 (26%)	0.52
Baseline platelet (K/μL, pre‐GCSF)	243 ± 58	219 ± 52	0.01	243 ± 46	212 ± 53	0.15
Recipient characteristics						
Age	50.5 ± 15.8	55.8 ± 13.1	0.02	61.9 ± 9.4	54.2 ± 13.6	0.08
Sex, *n* = 430						
Male	221 (57%)	25 (64%)	Reference	5 (63%)	20 (65%)	Reference
Female	170 (43%)	14 (36%)	0.36	3 (37%)	11 (35%)	0.92
Weight, kg	80.2 ± 23.4	83.8 ± 20.1	0.35	93.7 ± 13.9	81.3 ± 20.8	0.06
Recipient weight Δ (RDWD)	−5 ± 26.9	3.9 ± 23.9	0.04	20.4 ± 28	−0.1 ± 21.5	0.10
Weight Δ > 30 kg	31 (8%)	7 (18%)	0.03 OR 2.5 (CI: 1.04–6.26)	4 (50%)	3 (8%)	0.009 OR 9.3 (CI: 1.5–58)
Mobilization						
GCSF dosage (mcg/kg BW)						
10 mcg ABW	257/388 (66%)	26 (67%)	Reference	3 (37%)	23 (74%)	Reference
16 mcg IBW	119 (31%)	11 (28%)	0.81	3 (37%)	8 (26%)	0.33
Other^a^	12 (3%)	2	0.52	2 (25%)	0	—
CD34/μL, D1	91.8 ± 58.1	28.1 ± 22.1	< 0.0001	15.7 ± 9.6	30.6 ± 23.5	0.06
Post‐PLX	NA	NA		61.1 ± 47.6	22.5 ± 10.9	0.13
Platelet K/μL, D1	216 ± 54	195 ± 47	0.025	195 ± 37	195 ± 50	0.98
HPC collection						
Mean No. HPCC	1.3 ± 0.5	2.1 ± 0.5	< 0.0001	2.5 ± 0.5	2 ± 0.5	0.01
1 procedure	277 (70%)	4 (10%)	Reference	0	4	Reference
≥ 2 procedures	116 (30%)	35 (90%)	< 0.0001	8 (100%)	27 (87%)	0.43
Blood processed per procedure, L	16.9 ± 8.3	14.9 ± 8.3	0.16	11.9 ± 2.2	15.7 ± 8.1	0.026
#BV processed per procedure	3.3 ± 1	3.2 ± 1.5	0.46	3.0 ± 0.6	3.2 ± 1.7	0.71
Total volume blood processed, L	20.5 ± 8.1	34 ± 12	< 0.0001	31.4 ± 8.5	34.7 ± 12.9	0.40
Total #BV processed	4.2 ± 1.7	7.2 ± 2.9	< 0.0001	8.1 ± 2.5	7.0 ± 3.0	0.34
CD34 yields						
Day 1	8.8 ± 10.2	1.3 ± 0.47	< 0.0001	1.1 ± 0.36	1.3 ± 0.48	0.09
Final yield	10.2 ± 12.2	4.1 ± 2.4	< 0.0001	6.4 ± 3.0	3.6 ± 1.8	0.009
≥ 5 × 10^6^ CD34/kg	324 (83%)	12 (31%)	< 0.0001 OR = 10.6 (CI: 5.1–22)	6 (75%)	6 (19%)	0.002 OR = 12.5 (CI: 2–78)
≥ 4.5 × 10^6^ CD34/kg	344 (88%)	14 (36%)	< 0.0001 OR = 12.5 (CI: 6.1–25.7)	6 (75%)	8 (26%)	0.01 OR = 8.6 (CI: 1.4–51.7)

*Note:* Sex was not recorded for 2 of the 432 total recipients.

Abbreviations: BMI, body mass index (M^2^/kg); BV, donor blood volume; BW, donor body weight, where at actual body weight (ABW) or ideal body weight (IBW); CI, 95% confidence interval; D1, day 1 or first HPCC; GM, good mobilization; MRD, matched related donor; NA, not applicable; OR, odds ratio; PLX, plerixafor; PM, poor mobilization.

^a^ Other G‐CSF dosing ranged from 5 to 7.2 mcg/kg. No donor received more than 16 mcg/kg G‐CSF.

Compared to GM, PM were predominantly female (61% vs. 48%, *p* = 0.12), older (56 vs. 50 years, *p* = 0.004), with a higher percentage older than 60 years (49% vs. 25%, *p* = 0.001), and lower average baseline platelet counts prior to mobilization (219 vs. 243 k/μL, *p* = 0.01). PM had lower mean donor weights and obesity rates (Table 1). More importantly, PM were more likely to have a larger recipient–donor weight difference (RDWD, +19.4 vs. −5 kg, *p* = 0.01). An RDWD of 30 kg or greater was significantly associated with PM (18% vs. 8%, *p* = 0.035). In contrast, higher MRD weight and obesity favored GM.

PLX administration to MRD occurred in 4/8 centers with PM. A total of 8/432 MRD (1.8%) and 8/39 PM (20.5%) received PLX salvage. All PLX recipients were females donating for heavier recipients. In 50%, MRD receiving PLX had an RDWD ≥ 30 kg, which was significantly higher than PM who did not receive PLX (8%, *p* = 0.009). Among four MRD requiring three procedures, half received two doses of PLX and two received only a single dose. PLX salvage was used at the discretion of each site.

An increased CD34/μL and CD34 yield was observed in 7/8 PM after PLX. The mean peripheral CD34 count increased from 15.7 to 61.1/μL the following day. The mean CD34 yield/procedure increased from 1.26 to 3.86 × 10^6^/kg. PLX significantly increased the percent of PM who met the target CD34 yield of ≥ 4.5 and ≥ 5 × 10^6^/kg (75%; Table 1).

## 4 Discussion

Few publications address PM and use of PLX in allogeneic donors, with mobilization failure rates ranging from 2% to 4.6% [1, 2, 3, 4]. The MOBIL1 trial was a prospective study of PLX in MRD and matched unrelated donors (MUD) across three European study sites. Only 37/6293 (0.6%) donors required PLX for PM [1]. A 2021 retrospective Spanish multicenter study identified 30 MRD who received PLX [2]. Two more recent single‐center retrospective studies reported higher rates for PLX salvage of 4.1% and 4.6% [3, 4]. As with our study, factors associated with PLX salvage were female sex, lower BMI, and donation to a heavier recipient [2, 3, 4]. Large RDWDs and inferior CD34 yields have been reported in pediatric transplantation, with a donor‐recipient weight ratio < 0.75 significantly increasing the risk for multiple leukapheresis collections [5]. In bone marrow harvests, a RDWD of ≥ 20 kg is discouraged due to the risk for suboptimal yields [6]. In our study, a RDWD of 30 kg or more was strongly associated with PM and PLX salvage.

## 5 Conclusions

Risks of poor mobilization in MRD were older age, especially donors older than 60 years, lower baseline platelet counts, and heavier recipients. All PLX recipients were female. A RDWD greater than 30 kg doubled the risk for PM. PLX use in PM was low but safe, tripling the success rate for collection. This study adds evidence to the body of literature to support the use of PLX in allogeneic donors who are PM.

## Funding

The authors have nothing to report.

## Conflicts of Interest

The authors declare no conflicts of interest.

## Data Availability

The data that support the findings of this study are available from the corresponding author upon reasonable request.
